# Unique changes in the TCR repertoire of tumor-infiltrating lymphocytes underlie the synergy of radiotherapy with CTLA-4 blockade

**DOI:** 10.1186/2051-1426-2-S3-P141

**Published:** 2014-11-06

**Authors:** Karsten Pilones, Ryan Emerson, Silvia Formenti, Harlan Robins, Sandra Demaria

**Affiliations:** 1 NYU School of Medicine, New York, NY, USA; 2 Adative Biotechnologies, Seattle, WA, USA; 3 Fred Hutchinson Cancer Research, Seattle, WA, USA

## Background

Checkpoint blockade is increasingly becoming a valuable immunotherapeutic tool in the management of advanced malignancies. Monoclonal antibodies (mAb) that target CTLA-4 have significantly extended survival of patients with metastatic melanoma, however the number of responders remain low. We have previously shown in the 4T1 mouse tumor model that resistance to anti-CTLA-4 therapy can be overcome by concurrent local radiotherapy (RT) (Demaria et al 2005 Clin Can Res 11:728). Improved response was, in part, the result of radiation's ability to promote priming, and enhance homing of effector cytotoxic T cells to the tumor and their interactions with tumor cells (Matsumura et al 2008 J Immunol 181; Ruocco et al 2012 J Clin Invest 122:10). Here we used high throughput sequencing of T cell receptor (TCR) b chain to interrogate the breadth and depth of tumor infiltrating lymphocytes (TILs) repertoire changes in 4T1 tumors after treatment with anti-CTLA-4 therapy given in conjunction with radiotherapy.

## Methods

Balb/c mice were inoculated s.c. with 4T1 cells and thirteen days later, when tumors became palpable, randomly assigned to one of 4 treatment groups (n = 5 mice/group): control, RT alone, anti-CTLA-4 alone or RT+anti-CTLA-4. RT was given in 2 fractions of 12 Gy on days 13 and 14 post-tumor inoculation. Anti-CTLA-4 mAb (Clone 9H10) was given i.p. on days 15, 18 and 21. Tumors were harvested on day 22 for high throughput sequencing of TCRβ CDR3 regions performed using the ImmunoSEQ platform.

## Results

Data indicate distinct non-overlapping effects of the combination treatment. CTLA-4 blockade increased clonality and significantly expanded the top 5 most frequent clonotypes. On the other hand, radiation augmented TIL numbers and broadened their repertoire by selective expansion of the top 6-20 clones. Importantly, analysis of Vβ/Jβ usage landscape showed that the combined treatment generated the most dramatic change from baseline with the expansion of several unique Vβ/Jβ combinations not seen in tumor treated with RT or CTLA-4 blockade alone.

## Conclusions

Overall, data indicate that tumor rejection induced by RT+anti-CTLA-4 is associated with both quantitative and qualitative changes in the TIL repertoire. They also suggest that a broader repertoire of tumor-specific T cells may be critical for therapeutic success and is achieved by complementary effects of RT, which induces antigenic spread, and CTLA-4 blockade, which drives expansion of selected clones.

